# A Pilot Study to Evaluate the Dietary Intake of Adults Attending a Food Allergy Clinic, and Compare the Results Against the Final Diagnostic Outcome

**DOI:** 10.3389/falgy.2021.765029

**Published:** 2021-11-23

**Authors:** Isabel J. Skypala, Cecile F. Taylor, Anthony Pallister, Guy W. Scadding

**Affiliations:** ^1^Department of Allergy and Clinical Immunology, Royal Brompton & Harefield Hospitals, Guy's and St. Thomas' NHS Foundation Trust, London, United Kingdom; ^2^Department of Allergy and Clinical Immunology, Imperial College, London, United Kingdom; ^3^Department of Nutrition and Dietetics, Royal Free Hospital, London, United Kingdom; ^4^Department of Nutrition and Dietetics, Kettering General Hospital, Kettering, United Kingdom

**Keywords:** diet, nutrition, adult, allergy, intolerance

## Abstract

**Background:** The impact of poor diet on growth and development in children with a food allergy is well-recognized and researched. Food allergy is an increasing problem in adults, as are food intolerances. Another issue is the rising number of individuals adopting a vegetarian or vegan lifestyle. Studies evaluating the diet of adolescents and adults with food allergy against controls suggest their dietary intakes are similar. We wished to evaluate all patients attending a food allergy clinic to determine whether there were dietary and nutritional differences between those with a food allergy or a food intolerance.

**Methods:** All adults newly referred to a secondary care food allergy clinic in a UK hospital, in a 1-month period, were included in the study. Prior to their appointment, those who consented to take part had their height and weight documented and an assessment made of their habitual food intake. Their subsequent diagnosis was reviewed, and results for those with a confirmed diagnosis of food allergy were compared to those with a food intolerance or where the cause of symptoms was unknown.

**Results:** Thirty subjects were recruited, with full results available for 29 subjects, 15 of whom (52%) were diagnosed with a new/existing food allergy (FA). For the whole cohort, dietary intake was sufficient for protein, and most vitamins and minerals, whereas energy, carbohydrate, unsaturated fat and fiber intakes were well-below the reference range. Those with a FA had lower intakes of iron, zinc and vitamin B12 compared to those with no FA. In addition, iron and energy intakes were depleted in those avoiding nuts, and wheat avoidance was linked to a lower intake of riboflavin.

**Conclusion:** The results from this small exploratory study suggest that whilst the majority of nutrients in the diet are sufficient in adults presenting to the food allergy clinic, intakes of energy and fiber may be below the reference range. Those with a food allergy are more likely to have a reduced intake of iron, zinc and vitamin B12. As others have demonstrated, the exclusion of specific food groups can also affect nutritional intakes.

## Introduction

Avoidance of food for any reason is likely to impact on nutritional health. It has been well-reported that nutritional intakes in children with suspected or confirmed food allergy can be compromised by unsupervised dietary manipulation, restrictions due to cultural, religious or ethical observance (e.g., vegan diet), an aversion to many foods (the “fussy eater”), fear of introducing complementary foods and doubts over which foods to introduce (1, 2). Unsupervised milk avoidance has been linked to poor calcium intake, poor bone health, loss of bone mineral density and even to rickets (3–5). Children with multiple food allergies have a higher risk of impaired growth and an increased likelihood of inadequate nutrient intake than children without food allergies (6–8). Although growth is not an issue in adults, the elimination of staple foods can reduce the intake of nutrients, and so affect overall health. The prevalence of probable food allergy (FA) in European adults' ranges from 0.3 to 5.6%, but although doctor-diagnosed food allergy rates are stable, the prevalence of self-reported is increasing, with one survey finding that of the 10.8% of adults who thought they had at least one convincing food allergy, only 50% had a physician-diagnosed allergy (9–11).

It is clear that numerous adults concerned about food allergy or intolerance choose to avoid certain food groups, especially wheat (12). However, many will also be avoiding multiple foods due to suspected or actual food intolerance, such as those diagnosed with Pollen Food Syndrome (PFS) or Oral Allergy Syndrome. The nutritional status of adults with food allergy or intolerance could be further compromised if those individuals are following a vegetarian or vegan lifestyle, as diets may be low in vitamin B12, vitamin D, zinc, calcium and iron (13). A study on the nutritional profile of children and adults with a milk allergy reported that there were nutritional deficiencies, especially if more than one food was being avoided (14). In contrast, a recent study by Maslin et al. demonstrated little difference between the nutritional intakes of adolescents and adults with a food allergy compared to controls (15). We were interested in determining the nutritional profile of adults newly referred to an allergy clinic for a suspected food allergy. We hypothesized that these adults would have a reduced intake of one or more important nutrients regardless of their final diagnosis. The aim of the study was to compare the dietary intake of subjects with a final diagnosis of food allergy to those from individuals who had a food intolerance or no food allergy.

## Materials and Methods

This non-interventional observational study, undertaken at the Royal Brompton & Harefield NHS Foundation Trust (RBHT) in London, received ethical approval from Health and Care Research Wales Ethics Committee and HRS approval from RBHT. The primary objective of the study was to evaluate the nutritional intakes of adults with suspected food allergy and determine whether their intake of certain nutrients is below the recommended reference range, and also whether there are differences between those with a final diagnosis of food allergy compared to those with a food intolerance or no food allergy. The participants were adults aged 17 years or over with suspected food allergy attending their first appointment in the adult food allergy clinic at RBHT. All consecutive new patients were asked to take part in the study prior to their consultation unless they were unable to provide informed consent or had previously been investigated for a food allergy elsewhere. Prior to their consultation, new patients were interviewed by a student dietitian, who measured their height and weight, using a stadiometer and hospital weighing scales, and calculated their Body Mass Index (BMI). The dietitian then conducted a face-to-face interview, to determine their habitual dietary intake, including nutritional supplements, over a 7-day period using a standardized non-validated form, constructed based on published guidelines (see Appendix) (16). The information was then analyzed using dietary assessment software DietPlan6 (Forestfield Software Ltd, Horsham, UK). Unfortunately, this software was not accessible for all of the analysis, so another program, Nutritics UK version (Nutritics Ltd. Dublin, Ireland.), was utilized, however both programs are based on the same standardized food composition tables (17). The results from the nutritional intakes, excluding any nutrients from dietary supplements, were compared to UK Dietary Reference Values (DRV), or Estimated Average Requirement (EAR) as appropriate for each nutrient (17–19). Subjects were also asked to complete a standardized but non-validated diet history form, adapted from a published diet history tool based on expert opinion (See Appendix) (20). Following this, subjects were reviewed in the food allergy clinic and followed the standard pathway for food allergy diagnosis, which included taking an allergy focused diet and clinical history and allergy testing to appropriate foods. The diagnostic tests included skin prick tests (SPT) performed on the volar aspect of the forearm according to standardized techniques and international guidelines, with any resulting wheal ≥3 mm considered positive (21). The test reagents included aeroallergen and food extracts (ALK Abelló, Denmark), fresh foods and positive and negative control solutions (histamine hydrochloride 10 mg/ml and diluent). Tests also included a serum sample taken for the measurement of total Immunoglobulin E (IgE) antibody levels, and individual IgE antibody tests specific to foods or aeroallergens depending on the results of their SPT. The results of the nutritional intake assessment were analyzed immediately after the study was completed. After 18 months, the patients' clinical records were accessed and reviewed to record the test results and diagnostic outcome. The patients were then categorized as either having food allergy (FA), or no food allergy (no FA), with the latter including both those who were diagnosed with a food intolerance and those with no FA or food intolerance (FI).

### Statistical Analysis

This was an exploratory pilot study, evaluating differences between those with food allergy and those presenting with symptoms to foods but no food allergy, so it was difficult to calculate the sample size required. On the basis that there were two food allergy clinics per week providing ~8–10 new patients, and there was a time constraint of 4 weeks to undertake the study due to external factors, it was planned to collect data on 32–40 patients, and present the subsequent analysis as hypothesis generating. Statistical analysis was performed using the Statistical Package for the Social Sciences (SPSS version 25.0, IBM). The Shapiro-Wilk test was used to determine normality of continuous variables. As a tool for exploring possible differences between datasets, Chi-squared, Mann-Whitney or Kruskal Wallis tests were used. Differences in age, the intake of nutrients and percentage of recommended intake were measured using the Student's *t*-test.

## Results

### Patient Characteristics

**Figure 1:**
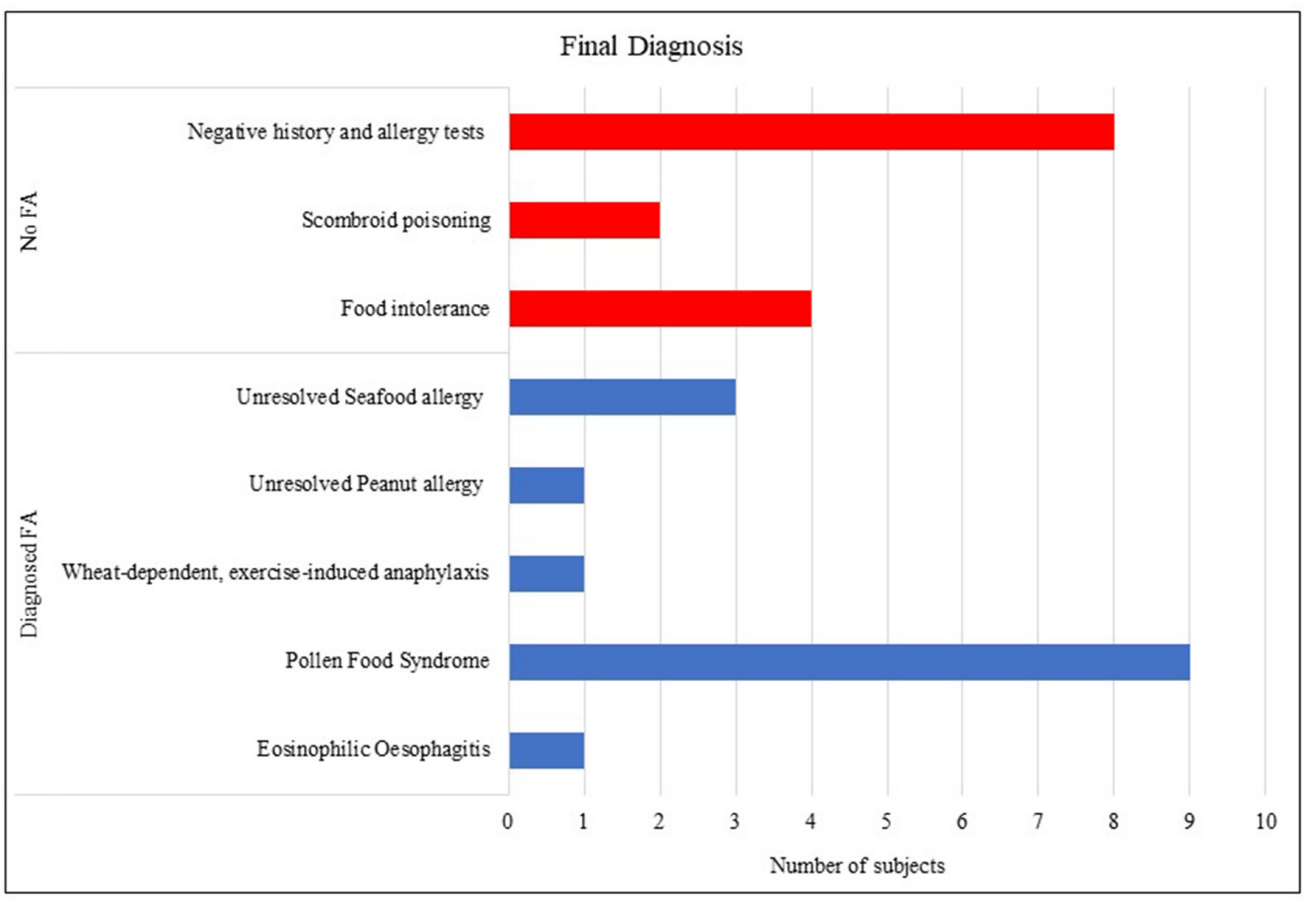
Final diagnosis of the study participants based on history, tests and expert evaluation.

Thirty Subjects Were Recruited but Data Was Incomplete for one Subject, so the Results Are Presented From 29 Subjects (18 Female), With a Mean age on Referral of 35.1 Years (Table 1). Data on Ethnicity, Although Collected, Was Incomplete for Some Patients and so not Reported Here. Of the 29 Subjects, 22 (76%) Reported Symptoms to Foods Developing in Adult Life (Aged 18–60 Years), 4 in Adolescence (Aged 15–17 Years) and 3 in Early Childhood (Aged 2–4 Years). Fifteen Subjects (52%) Had a Final Diagnosis of FA, 10 (45%) of Those With Adult-Onset Symptoms, 2 (50%) who Developed Symptoms in Teenage Years and all 3 (100%) of Those With Onset in Childhood. Those With Confirmed FA Were Most Likely to Have a new Diagnosis of Pollen Food Syndrome (PFS) (60%); three Subjects Had Unresolved Allergy From Childhood or Adolescence (27%), and one Each With Newly Diagnosed Wheat-Dependent, Exercise-Induced Anaphylaxis and Eosinophilic Esophagitis, a non-IgE-Mediated Food Allergy (see Figure 1). There Were no Differences Between Those Diagnosed With a FA and Those With no FA for age of Onset, Gender, Concomitant Asthma, Eczema or Current Food Allergy (Table 1). Those With a Final Diagnosis of FA Had a Lower Mean BMI of 21.7, Compared to 26.2 for the Group With no FA, but This Was not Significant. Reported Allergic Rhinitis Was Significantly More Likely to be Reported by Those With a FA, who Were Also Less Likely to Have Other Medical Conditions (Table 1).

**Table 1:** Demographic details.

**Variable**	**Whole cohort**	**FA**	**No FA**	***P*-value**
	**Mean (median, range)**	**Mean (median, range)**	**Mean (median, range)**	
Number of subjects	29	15	14	
Age on referral	35.1 (29, 18–62)	32.4 (28, 22–62)	38.5 (36, 18–61)	0.193^*^
Age of onset	27.8 (24, 2–60)	23.5 (23, 2–60)	32 (31, 15–58)	0.094^*^
Gender	11 male (40%)	8 male (53%)	3 male (21%)	0.077^±^
	18 female (60%)	7 female (47%)	11 female (79%)	
BMI	24.19 9 (17.5–49.9)	21.7	26.2	0.064^*^
Asthma	14 (48%)	9 (64%)	5 (36%)	0.191^±^
Eczema	10 (34%)	5 (50%)	5 (50%)	1.000^±^
Allergic rhinitis	22 (76%)	14 (64%)	8 (36%)	**0.023** ^±^
Other reported medical condition	9 (31%)	2 (22%)	7 (78%)	0.033^±^

* *Student t-test, ± Chi square test*.

### Symptoms, Foods Avoided and Test Results

The analysis of the symptom history revealed that the most frequently reported symptoms were oropharyngeal symptoms (80% of the cohort), and skin symptoms of itching, flushing or hives (55.2%), but there were no significant differences in reported symptoms or speed of symptom onset between those who were diagnosed with FA and those who were not. In terms of reported food triggers, the most frequently reported were fruit (10/29), wheat (9/29), tree nuts (8/29), peanuts (7/29) and crustaceans (7/29), with wheat significantly more likely to be reported by those with no FA, whereas those with a FA were significantly more likely to report reactions to mollusks, tree nuts and apple (Table 2). Of the foods being avoided at the time of the consultation, either due to allergy or other reasons, 55% were avoiding tree nuts, 41% fruit, 34% seafood, 34% vegetables and 31% wheat, with significantly more subjects with FA avoiding tree nuts, and those with no FA avoiding wheat (Table 2; Figure 1). There was no difference between groups for the number of food triggers reported, or the number of food groups being avoided. Individual food SPT results were not compared between groups as not every patient had every test performed, however, 15/15 of those with FA and 12/15 with no FA were tested to grass (*Phelum pratense*) and silver birch (*Betula verrucosa*). Subjects with a final diagnosis of FA were significantly more likely to have a positive test to both grass (14/15, 93%) and birch (12/15, 80%), than those with no FA (4/12, 33%—grass, *p* = 0.002) and (1/12, 8%—birch, *p* = < 0.001).

**Table 2:** Foods reported to provoke reactions or being avoided.

	**Reported symptoms to foods**				**Avoiding foods**		
	**FA (15)**	**No FA (14)**	***P-*value**		**FA (15)**	**No FA (14)**	***P-*value**
Milk	3	3	0.924	Milk	2	5	0.129
Egg	1	2	0.501	Egg	2	1	0.584
Fish	2	1	0.584	Seafood	7	3	0.153
Crustaceans	5	2	0.231	Wheat	2	7	**0.033**
Molluscs	6	0	**0.008**	Fruit	8	4	0.176
Wheat	3	6	0.184	Vegetables	4	6	0.359
Apple	6	1	**0.039**	Peanuts	4	3	0.742
Kiwi	4	1	0.164	Seeds	1	1	0.960
Any fruit	7	3	0.153	Tree nuts	11	5	**0.042**
Vegetables	1	2	0.501	Meat	3	6	0.184
Soy	0	1	0.292				
Peanuts	4	3	0.742				
Seeds	2	1	0.584				
Tree nuts	7	1	**0.017**				

### Nutritional Intakes

**Figure 2:**
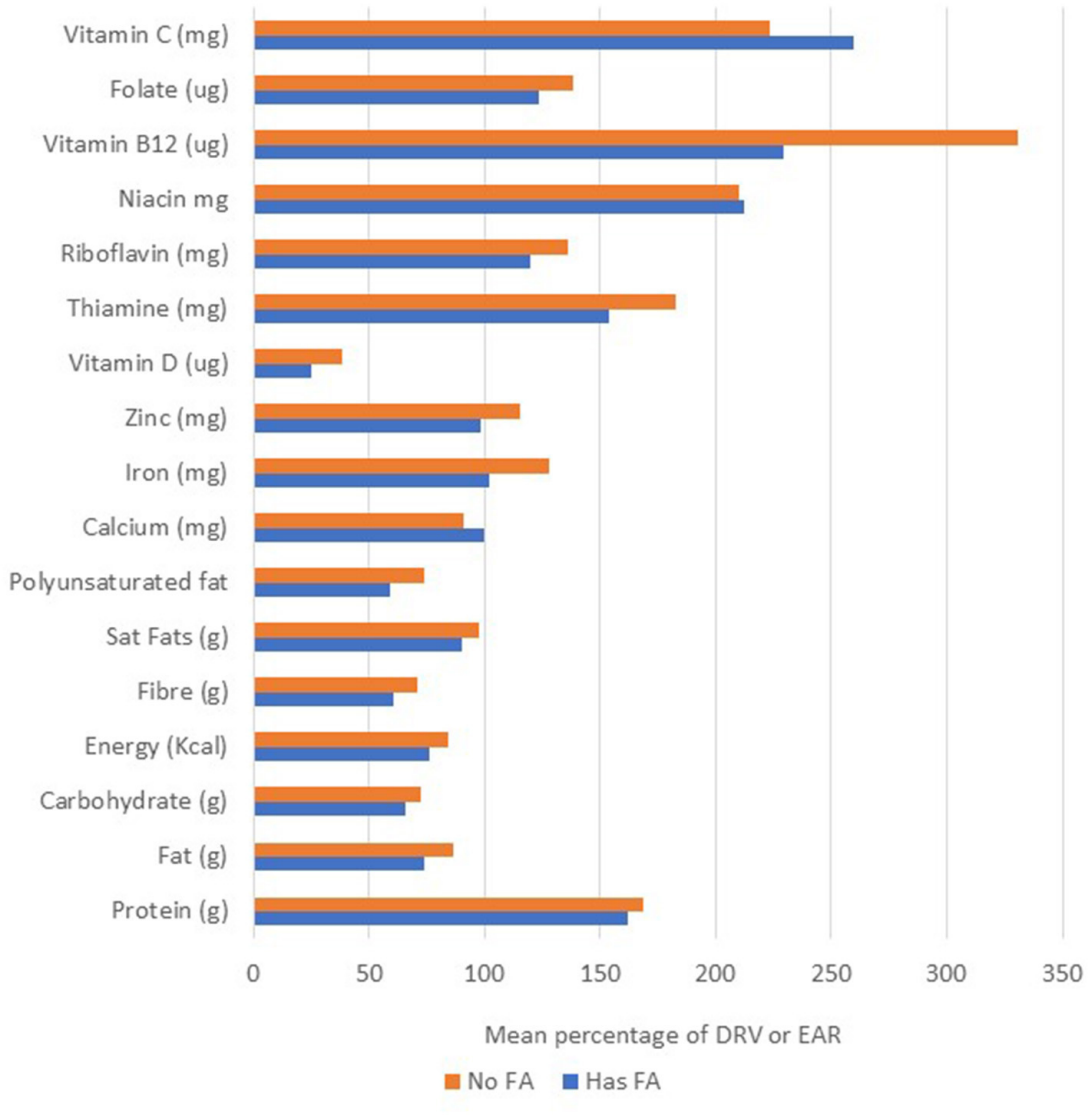
Mean percentage dietary reference value for all nutrients.

In terms of their nutritional intake there were few differences between groups, although those with a FA had overall greater intakes of protein and niacin when compared to those with no FA, but a significantly lower intake of iron (Table 3; Figure 2). These data were analyzed to determine on an individual basis what percentage of the DRV or EAR each participant achieved for each individual nutrient. The analysis presented is for the nutrient intake from food and beverages only, with any intake from nutritional supplements (where consumed) not included. The collective results demonstrated that although there were no significant differences between the groups, those with a food allergy were more likely to have lower intakes of iron, zinc and Vitamin B12, and in the whole cohort there was considerably <100% achievement of the DRV for carbohydrate, energy, fiber and Vitamin D (Table 3). An analysis of the number of subjects with levels below the reference range revealed that in addition to low intakes of carbohydrate, energy, fiber and vitamin D, over a third of both those with or with no FA had intakes of the B vitamins, folate and riboflavin below the reference range, and the majority (67%) of those with a food allergy also had a low level of iron (Table 3). An analysis of the nutritional intake of the whole cohort assessed whether subjects avoiding specific foods were more likely to have intakes below the RDI for individual nutrients (Table 4). The results revealed that the avoidance of tree nuts was linked to a low intake of energy and iron, wheat avoidance with a reduced intake of riboflavin and fruit avoidance with a low intake of iron (Table 4). Two-thirds of those avoiding meat had a significantly lower intake of energy (Table 4). Eight patients (1 FA, 7 no FA) in the cohort (28%) were taking nutritional supplements, including multi-vitamin preparations (5/8), fish oil (1/8), glucosamine (1/8), and one where the supplement was not recorded. There was no significant difference in the mean number of nutrients below the RDI, between those taking supplements and those who did not, but there was a correlation between diagnosis (has a FA or no FA) and the use of supplements (Spearman *r* = 0.48448, *p* = 0.00774).

**Table 3:** Results of the dietary assessment, showing the nutrient intakes for the whole cohort, and also for the group with food allergy, compared to those with no food allergy.

	**Mean nutritional intake**			
	**Whole cohort**	**FA**	**No FA**	***P*-value**
	**(range)**			**(*t*-test)**
Protein (g)	81.3 (28.4–121.4)	89.8	72.1	**0.034**
Fat (g)	67.1 (31.6–106)	65.0	69.2	0.55
Carbohydrate (g)	200 (75.3–295.7)	201.0	198.0	0.879
Energy (Kcal)	1761 (777–2,243)	1805.0	1714.0	0.462
Fiber (g)	20.2 (9.9–34.3)	18.4	22.1	0.141
Saturated Fat (g)	24.6 (6.4–52.4)	24.1	25.1	0.779
Polyunsaturated Fat (g)	10.17 (3–16.6)	9.5	10.9	0.366
Calcium (mg)	670 (284–1148)	669.0	671.0	0.982
Iron (mg)	12.7 (4.2–29.6)	10.5	15.1	**0.027**
Zinc (mg)	8.5 (4.1–13.4)	8.2	8.7	0.543
Vitamin D (ug)	3.1 (0–12.5)	3.8	2.3	0.201
Thiamine (mg)	1.5 (0.8–3.3)	1.4	1.4	0.876
Riboflavin (mg)	1.5 (0.4–3.4)	1.5	1.4	0.866
Niacin mg	30.8 (3.5–53.0)	36.8	24.3	**0.016**
Vitamin B12 (ug)	4.1 (1–9.5)	4.1	4.0	0.949
Folate (ug)	260 (120–542)	266.0	255.0	0.792
Vitamin C (mg)	97.7 (6.5–388)	82.0	114.0	0.227

**Table 4:** The number of patients avoiding a specific food who have intakes below the RDI for individual nutrients.

**Below RDI for**	**Food being avoided (no subjects)**							
	**Milk (7)**	**Wheat (9)**	**Peanuts (7)**	**Tree nuts (16)**	**Seafood (10)**	**Fruit (12)**	**Vegetables (10)**	**Meat (9)**
Protein	0 (0.566^*^)	1 (0.129)	0 (0.566)	1 (0.359)	0 (0.460)	0 (0.393)	1 (0.460)	1 (0.129)
Fat	4 (0.096)	7 (0.891)	6 (0.631)	13 (0.775)	8 (0.947)	10 (0.653)	8 (0.947)	7 (0.891)
Carbohydrate	7 (0.566)	8 (0.129)	7 (0.566)	16 (0.259)	10 (0.460)	12 (0.393)	10 (0.460)	9 (0.495)
Energy	6 (0.965)	8 (0.779)	7 (0.224)	**16 (0.017)**	9 (0.667)	11 (0.474)	10 (0.118)	**6 (0.041)**
Fiber	5 (0.069)	7 (0.159)	6 (0.694)	15 (0.422)	10 (0.184)	11 (0.765)	9 (0.965)	8 (0.928)
Saturated fat	4 (0.438)	6 (0.858)	5 (0.872)	10 (0.404)	7 (0.930)	8 (0.822)	9 (0.076)	7 (0.491)
Polyunsaturated fat	5 (0.193)	7 (0.377)	7 (0.224)	**16 (0.017)**	9 (0.667)	11 (0.474)	9 (0.667)	8 (0.779)
Calcium	5 (0.321)	7 (0.101)	4 (0.904)	9 (0.897)	5 (0.684)	7 (0.774)	5 (0.684)	**2 (0.017)**
Iron	2 (0.159)	4 (0.599)	4 (0.742)	**11 (0.042)**	4 (0.359)	**9 (0.035)**	6 (0.518)	4 (0.599)
Zinc	2 (0.430)	4 (0.822)	2 (0.430)	8 (0.296)	4 (0.913)	4 (0.460)	5 (0.494)	5 (0.298)
Vitamin D	7 (0.566)	9 (0.495)	7 (0.566)	16 (0.259)	10 (0.460)	11 (0.226)	10 (0.460)	8 (0.129)
Thiamine	0 (0.566)	1 (0.129)	0 (0.566)	1 (0.359)	0 (0.460)	0 (0.393)	0 (0.460)	1 (0.129)
Riboflavin	4 (0.229)	**6 (0.032)**	2 (0.588)	7 (0.474)	4 (0.868)	6 (0.260)	5 (0.331)	3 (0.732)
Niacin	1 (0.376)	2 (0.29)	0 (0.408)	1 (0.879)	0 (0.288)	1 (0.798)	1 (0.632)	**2 (0.029)**
Vitamin B12	1 (0.694)	2 (0.159)	1 (0.694)	2 (0.672)	1 (0.965)	0 (0.124)	1 (0.965)	1 (0.928)
Folate	3 (0.593)	4 (0.449)	1 (0.197)	6 (0.705)	2 (0.234)	6 (0.140)	4 (0.650)	4 (0.449)
Vitamin C	0 (0.224)	0 (0.148)	2 (0.193)	4 (0.052)	3 (0.066)	3 (0.141)	3 (0.066)	2 (0.377)

* *Chi square*.

## Discussion

The results from this pilot study found that most adults attending the food allergy clinic had low reported intakes of major nutrients including carbohydrate, fat and fiber. Although protein and vitamin intakes were generally above the recommended dietary reference range, iron, zinc, folate and riboflavin intakes were often low, especially in those with a diagnosis of FA. Nutritional disorders have often been cited as being of particular concern in children with food allergy, but there are few if any papers which have investigated the nutritional intakes of adults with food allergy or intolerance (22–24). It is now clear that nutrition in early life is a key to preventing food allergy, with early introduction of key allergenic foods and the diversity of the diet both being important (25, 26). In food-allergic children, it is the exclusion of milk which most often signals an issue with nutritional intake (27). A study by Kim et al. which included 225 children and adults aged 1–65 years with atopic dermatitis, found that those with a documented allergy to milk had reduced intakes of calcium, zinc and vitamin B12 (14). Our cohort was too small to make a direct comparison, but there was no link between the exclusion of milk and an intake of any nutrients significantly below the RDI. Also, milk was not so frequently excluded as other foods such as tree nuts.

The greatest number of nutritional deficiencies in Kim's study was linked to the exclusion of wheat and soy, and included calcium, iron, zinc, riboflavin, vitamin B6, niacin. Our data also found that riboflavin and niacin levels were significantly more likely to be below the RDI in those avoiding wheat and/or soy. The exclusion of wheat through choice rather than necessity has become highly prevalent in adults. A recent large survey in Australia reported that 24% of participants were avoiding wheat (gluten), half of whom were doing so as they perceived it to be better for their general health (12). Similarly, a Canadian survey found that 1.9% of Canadians follow a gluten-free diet, and those who did so had lower intakes of iron, calcium, folate, B12 and Vitamin D than those who were eating wheat (28). In our cohort, wheat was significantly more likely to be avoided by those with no FA, which is consistent with findings from other studies of individuals with conditions such as irritable bowel syndrome (29). Of the nine subjects avoiding wheat in our study, the vast majority had intakes of energy, carbohydrate, fiber, calcium and vitamin D below the recommended level, and a significantly lower intake of riboflavin compared to those not avoiding wheat. Given the low numbers, a larger study is needed to verify these findings, but it is clear from other studies that the exclusion of wheat and other grains can result in nutritional deficiency, especially since gluten-free products often contain less fiber and more fat and sugar than comparable wheat-based products (30). It is also known that gluten-free diets are often high in protein and low in complex carbohydrates, which was the case for all of our cohort, whether they were avoiding wheat or other foods (31, 32).

With regard to nutritional supplements, Maslin and colleagues reported that 27% of adults in their study took dietary supplements, with was no difference between the cases and controls. Those adults with a FA had a higher intake of folate and zinc but this difference disappeared when supplement data was removed. Our data showed that supplement use was not linked to the number of foods being excluded or the number of nutrients below the RDI. However, what was significant was that those not diagnosed with a FA were more likely to take supplements, which may reflect a greater focus on their general health and well-being. Kim's study highlighted the fact that the greater the number of foods being avoided, the more likely a nutritional deficiency is to occur. Our data showed that overall, the median number of foods being excluded was 2 and the median number of nutrients below the reference range was 8, with no significant difference between those with or without a FA. There was a correlation between the number of foods excluded and the number of people with nutrient intakes below the reference range, but only for the group with no FA (Spearman *r* = 0.5074, *p* = 0.064). The challenge of ensuring dietary and nutritional needs are met in those with food allergy is exacerbated by increased exclusion of foods due to lifestyle choices such as vegetarianism and veganism (13). In the cohort studied, 9/29 (31%) were avoiding meat, two of whom were also avoiding fish. There were few major nutritional differences between those who did and did not eat meat; those who avoided meat were proportionally less likely to be deficient in energy and more likely to achieve their DRV for calcium, but less likely to meet their requirement for Niacin that those who ate meat (Table 4). There was a greater proportion of people avoiding meat in the no FA group (6/14) compared to the FA group (3/15) but this was no significant (Table 2).

The avoidance of major food groups is of particular concern for those diagnosed with PFS who may react to multiple foods. It was this group of individuals who were most likely to present with symptoms in adult life; 60% of those with a diagnosis of FA had PFS. This pollen-related food allergy is an increasing problem worldwide, affecting up to 70% of adults with seasonal allergic rhinitis, with a prevalence of 2% in unselected UK adults (33, 34). Those affected experience oropharyngeal symptoms to one or more raw plant foods, especially fruits and tree nuts (35). Often multiple foods are involved and although the symptoms are usually mild, they can range in severity, with a small percentage suffering from anaphylaxis (36). A sub analysis demonstrated that those with PFS were significantly more likely to report a greater number of foods caused reactions (*p* = 0.004). It is interesting to note that although principally avoiding fruits and nuts, 89% of those diagnosed with PFS did not meet their individual RDI for iron, compared with 33% for those with a non-PFS FA, and 36% of those with no FA (*p* = 0.027). The exclusion of tree nuts, common in those with PFS, was also linked to low intakes of vitamin C and significantly lower intakes of energy and iron. Due to its usually mild nature and the fact that it is perceived to only occur in adults, PFS is often not considered to be likely to affect nutritional status. However, our data, and the fact that many sufferers exclude multiple fruits, vegetables and tree nuts, means that nutritional consequences in these individuals needs to be addressed by individual counseling (35).

This pilot study suggests that individuals attending a food allergy clinic are often avoiding several food groups which may impact on their nutritional status. These data need to be interpreted carefully, due to the small number of subjects and methodology utilized to collect dietary intake data. The inclusion of data from a healthy control group would enhance the findings of a larger study, to determine whether our data is generalizable to a larger group of subjects. We could not find any published studies evaluating the nutritional intake of food allergic individuals against those avoiding foods due to a food intolerance or for other reasons, to enable us to calculate a suitable sample size. Hopefully these data will be useful in providing a future sample size, especially since our data also showed the proportion of adults presenting in the food allergy clinic who subsequently are diagnosed with a food allergy was ~50%, as has been demonstrated in other studies (11). Other than the lack of a sample size calculation, there is also a danger of underreporting of dietary intake, an acknowledged issue with dietary intake studies, although this applies more to self-completed food frequency questionnaires (37, 38). In our study, the dietary details were ascertained from patients by an individual who was well-trained in the collection of such data.

A larger study should include more standardized methods of determining nutritional intake, and also address the issue of reporting bias. The design of this study was to capture dietary intake data from a group of adults referred for a suspected food allergy. In order to provide more robust data, a control group of matched healthy adults would be included in a larger study, but the design would continue to include all patients attending the clinic to further determine similarities and differences between those with a diagnosed IgE-mediated food allergy, those with a diagnosed intolerance and those diagnosed with no FA or FI. An evaluation of nutritional status, especially iron, folate, B12 and calcium would also be useful to include in further studies. It is particularly noteworthy that whilst most participants had intakes of calcium, B vitamins, and vitamin C that were within the normal range, intakes of energy, carbohydrate, fiber, iron and vitamin D were low, which needs further investigation. Also, those avoiding milk did not have significantly lower intakes than those avoiding other foods, which may indicate that many milk substitutes are now sufficiently fortified to ensure calcium intakes are not compromized. Although this study has reported on the major nutrients, the diet as a whole is important for the optimization of the gut microbiome and also for promoting anti-inflammatory responses (39). Fruits, vegetables, and whole grain cereals, together with omega 3 fatty acids and fatty fish are some of the most important anti-inflammatory foods or food components (40). More research is needed to assess the effect of excluding these foods from the diet.

Studies have suggested that 50% of adults with a reported food allergy are likely to have a physician-diagnosed food allergy (11). Our snapshot of new patients attending the food allergy clinic accords with this, with just over 55% having a confirmed food allergy. However, overall, the level of food avoidance and nutritional compromise was not significantly different between those who did and did not have a food allergy. It is therefore important to ensure that regardless of the diagnosis, patients attending an allergy clinic who are avoiding multiple foods should have access to nutritional counseling, so they can enjoy a healthy and nutritionally complete diet.

## Conclusion

The nutritional aspects of food allergy have assumed a much greater importance in the last few years. Much work has been undertaken in pregnancy and early life to determine how nutritional status affects the outcomes of allergic disease. Few studies however have addressed the issue of whether dietary intakes in adults are nutritionally sound, and whether there are differences between those with a diagnosed food allergy and those who have a food intolerance or no food allergy. The numbers of adults who are excluding foods from their diet is increasing, whether this is due to perceived health reasons or because of concerns that particular foods are provoking symptoms. Our study demonstrates that the intake of some key nutrients such as fiber, iron and Vitamin D are low in all subjects presenting to the allergy clinic with suspected food allergy. The findings highlight the need for dietary counseling for such individuals, especially if they are excluding more than one food.

## Data Availability Statement

The data presented in this article is not readily available. Requests to access the data should be directed to i.skypala@rbht.nhs.uk.

## Ethics Statement

The studies involving human participants were reviewed and approved by Health and Care Research Wales Ethics Committee. The patients/participants provided their written informed consent to participate in this study.

## Author Contributions

IS wrote the protocol, applied for ethical approval, interviewed some of the patients, analyzed the results, and wrote the manuscript. CT interviewed some of the patients, undertook some of the dietary analysis and reviewed, and commented on the paper. AP undertook some of the dietary analysis and commented on the paper. GS was the named consultant for the clinic, had input into the protocol and design of the study, and commented on the paper. All authors contributed to the article and approved the submitted version.

## Conflict of Interest

The authors declare that the research was conducted in the absence of any commercial or financial relationships that could be construed as a potential conflict of interest.

## Publisher's Note

All claims expressed in this article are solely those of the authors and do not necessarily represent those of their affiliated organizations, or those of the publisher, the editors and the reviewers. Any product that may be evaluated in this article, or claim that may be made by its manufacturer, is not guaranteed or endorsed by the publisher.

## References

[1] Groetch M Henry M Feuling MB Kim J. Guidance for the nutrition management of gastrointestinal allergy in pediatrics. J Allergy Clin Immunol Pract. (2013) 1:323–31. 10.1016/j.jaip.2013.05.002 24565537

[2] Mailhot G Perrone V Alos N Dubois J Delvin E Paradis L. Cow's milk allergy and bone mineral density in prepubertal children. Pediatrics. (2016) 137:e20151742. 10.1542/peds.2015-1742 27244780

[3] Doulgeraki AE Manousakis EM Papadopoulos NG. Bone health assessment of food allergic children on restrictive diets: a practical guide. J Pediatr Endocrinol Metab. (2017) 30:133–9. 10.1515/jpem-2016-0162 28099128

[4] Yu JW Pekeles G Legault L McCusker CT. Milk allergy and vitamin D deficiency rickets: a common disorder associated with an uncommon disease. Ann Allergy Asthma Immunol. (2006) 96:615–9. 10.1016/S1081-1206(10)63558-2 16680934

[5] Meyer R De Koker C Dziubak R Venter C Dominguez-Ortega G Cutts R. Malnutrition in children with food allergies in the UK. J Hum Nutr Diet. (2014) 27:227–35. 10.1111/jhn.12149 23937486

[6] Flammarion S Santos C Guimber D Jouannic L Thumerelle C Gottrand F. Diet and nutritional status of children with food allergies. Pediatr Allergy Immunol. (2011) 22:161–5. 10.1111/j.1399-3038.2010.01028.x 20561235

[7] Vieira MC Morais MB Spolidoro JV Toporovski MS Cardoso AL Araujo GT. A survey on clinical presentation and nutritional status of infants with suspected cow' milk allergy. BMC Pediatr. (2010) 10:25. 10.1186/1471-2431-10-25 20416046 PMC2873518

[8] Meyer R De Koker C Dziubak R Godwin H Dominguez-Ortega G Chebar Lozinsky A. The impact of the elimination diet on growth and nutrient intake in children with food protein induced gastrointestinal allergies. Clin Transl Allergy. (2016) 6:25. 10.1186/s13601-016-0115-x 27418957 PMC4944436

[9] Lyons SA Burney PGJ Ballmer-Weber BK Fernandez-Rivas M Barreales L Clausen M. Food allergy in adults: substantial variation in prevalence and causative foods across Europe. J Allergy Clin Immunol Pract. (2019) 7:1920–8.e11. 10.1016/j.jaip.2019.02.044 30898689

[10] Verrill L Bruns R Luccioli S. Prevalence of self-reported food allergy in U.S. Adults: 2001, 2006, and 2010. Allergy Asthma Proc. (2015) 36:458–67. 10.2500/aap.2015.36.3895 26453524 PMC4623408

[11] Gupta RS Warren CM Smith BM Jiang J Blumenstock JA Davis MM. Prevalence and severity of food allergies among US adults. JAMA Netw Open. (2019) 2:e185630. 10.1001/jamanetworkopen.2018.5630 30646188 PMC6324316

[12] Potter M Jones MP Walker MM Koloski NA Keely S Holtmann G. Incidence and prevalence of self-reported non-coeliac wheat sensitivity and gluten avoidance in Australia. Med J Aust. (2020) 212:126–31. 10.5694/mja2.50458 31909482

[13] Protudjer JLP Mikkelsen A. Veganism and paediatric food allergy: two increasingly prevalent dietary issues that are challenging when co-occurring. BMC Pediatr. (2020) 20:341. 10.1186/s12887-020-02236-0 32650748 PMC7350184

[14] Kim J Kwon J Noh G Lee SS. The effects of elimination diet on nutritional status in subjects with atopic dermatitis. Nutr Res Pract. (2013) 7:488–494. 10.4162/nrp.2013.7.6.488 24353835 PMC3865272

[15] Maslin K Venter C MacKenzie H Vlieg-Boerstra B Dean T Sommer I. Comparison of nutrient intake in adolescents and adults with and without food allergies. J Hum Nutr Diet. (2018) 31:209–17. 10.1111/jhn.12495 28707418

[16] Gandy J. Manual of Dietetic Practice. Wiley Blackwell. (2019). Available online at: https://www.wiley.com/en-gb/Manual+of+Dietetic+Practice,+6th+Edition-p-9781119235910 (accessed December 08, 2021).

[17] McCance RA Widdowson EM. Institute of Food Research (Great Britain), Public Health England, & Royal Society of Chemistry (Great Britain). McCance and Widdowson's the Composition of Foods. (2015). Available online at: https://www.gov.uk/government/publications/composition-of-foods-integrated-dataset-cofid (accessed December 08, 2021).

[18] Shim JS Oh K Kim HC. Dietary assessment methods in epidemiologic studies. Epidemiol Health. (2014) 36:e2014009. 10.4178/epih/e2014009 25078382 PMC4154347

[19] Dietary reference values for food energy and nutrients for the United Kingdom. Report of the panel on dietary reference values of the committee on medical aspects of food policy. Rep Health Soc Subj. (1991) 41:1–210. 1961974

[20] Skypala IJ de Jong NW Angier E Gardner J Kull I Ryan D. Promoting and achieving excellence in the delivery of integrated allergy care: the European Academy of Allergy & Clinical Immunology competencies for allied health professionals working in allergy. Clin Transl Allergy. (2018) 8:31. 10.1186/s13601-018-0218-7 30151118 PMC6102852

[21] Muraro A Werfel T Hoffmann-Sommergruber K Roberts G Beyer K Bindslev-Jensen C. EAACI food allergy and anaphylaxis guidelines: diagnosis and management of food allergy. Allergy. (2014) 69:1008–25. 10.1111/all.12429 24909706

[22] Meyer R. Nutritional disorders resulting from food allergy in children. Pediatr Allergy Immunol. (2018) 29:689–704. 10.1111/pai.12960 30044008

[23] Venter C Mazzocchi A Maslin K Agostoni C. Impact of elimination diets on nutrition and growth in children with multiple food allergies. Curr Opin Allergy Clin Immunol. (2017) 17:220–6. 10.1097/ACI.0000000000000358 28323676

[24] Skypala IJ McKenzie R. Nutritional issues in food allergy. Clin Rev Allergy Immunol. (2019) 57:166–78. 10.1007/s12016-018-8688-x 29766369

[25] Fleischer DM Chan ES Venter C Spergel JM Abrams EM Stukus D. A consensus approach to the primary prevention of food allergy through nutrition: guidance from the American academy of allergy, asthma, and immunology; American college of allergy, asthma, and immunology; and the canadian society for allergy and clinical immunology. J Allergy Clin Immunol Pract. (2021) 9:22–43.e4. 10.1016/j.jaip.2020.11.002 33250376

[26] D'Auria E Abrahams M Zuccotti GV Venter C. Personalized nutrition approach in food allergy: is it prime time yet? Nutrients. (2019) 11:359. 10.3390/nu11020359 30744105 PMC6412250

[27] Maslin K Oliver EM Scally KS Atkinson J Foote K Venter C. Nutritional adequacy of a cows' milk exclusion diet in infancy. Clin Transl Allergy. (2016) 6:20. 10.1186/s13601-016-0109-8 27257475 PMC4890506

[28] Mudryj AN Waugh AK Slater JJ Duerksen DR Bernstein CN Riediger ND. Nutritional implications of dietary gluten avoidance among canadians: results from the 2015 Canadian community health survey. Br J Nutr. (2021) 126:738–46. 10.1017/S000711452000450X 33172514

[29] Patel K Vila-Nadal G Shah J Shamji MH Swan L Durham SR. Is pollen-food syndrome a frequent comorbidity in adults with irritable bowel syndrome? Allergy. (2020) 75:1780–3. 10.1111/all.14209 31999843

[30] Babio N Lladó Bellette N Besora-Moreno M Castillejo G Guillén N Martínez-Cerezo F. A comparison of the nutritional profile and price of gluten-free products and their gluten-containing counterparts available in the Spanish market. Nutr Hosp. (2020) 37:814–22. 10.20960/nh.03016 32686439

[31] Suárez-González M Bousoño García C Jiménez Treviño S Iglesias Cabo T Díaz Martín JJ. Influence of nutrition education in paediatric coeliac disease: impact of the role of the registered dietitian: a prospective, single-arm intervention study. J Hum Nutr Diet. (2020) 33:775–85. 10.1111/jhn.12800 32790023

[32] Gładyś K Dardzińska J Guzek M Adrych K Małgorzewicz S. Celiac dietary adherence test and standardized dietician evaluation in assessment of adherence to a gluten-free diet in patients with celiac disease. Nutrients. (2020) 12:2300. 10.3390/nu12082300 32751809 PMC7468751

[33] Muluk NB Cingi C. Oral allergy syndrome. Am J Rhinol Allergy. (2018) 32:27–30. 10.2500/ajra.2018.32.4489 29336286

[34] Skypala IJ Bull S Deegan K Gruffydd-Jones K Holmes S Small I. The prevalence of PFS and prevalence and characteristics of reported food allergy; a survey of UK adults aged 18-75 incorporating a validated PFS diagnostic questionnaire. Clin Exp Allergy. (2013) 43:928–40. 10.1111/cea.12104 23889246

[35] Carlson G Coop C. Pollen food allergy syndrome (PFAS): a review of current available literature. Ann Allergy Asthma Immunol. (2019) 123:359–65. 10.1016/j.anai.2019.07.022 31376490

[36] Kim M Ahn Y Yoo Y Kim DK Yang HJ Park HS. Clinical manifestations and risk factors of anaphylaxis in pollen-food allergy syndrome. Yonsei Med J. (2019) 60:960–8. 10.3349/ymj.2019.60.10.960 31538431 PMC6753338

[37] Burrows TL Ho YY Rollo ME Collins CE. Validity of dietary assessment methods when compared to the method of doubly labeled water: a systematic review in adults. Front Endocrinol. (2019) 10:850. 10.3389/fendo.2019.00850 31920966 PMC6928130

[38] Garden L Clark H Whybrow S Stubbs RJ. Is misreporting of dietary intake by weighed food records or 24-hour recalls food specific? Eur J Clin Nutr. (2018) 72:1026–34. 10.1038/s41430-018-0199-6 29789710

[39] Galland L. Diet and inflammation. Nutr Clin Pract. (2010) 25:634–40. 10.1177/0884533610385703 21139128

[40] Shivappa N Steck SE Hurley TG Hussey JR Hébert JR. Designing and developing a literature-derived, population-based dietary inflammatory index. Public Health Nutr. (2014) 17:1689–96. 10.1017/S1368980013002115 23941862 PMC3925198

