# Supplementary material for: A Pilot Study to Evaluate the Dietary Intake of Adults Attending a Food Allergy Clinic, and Compare the Results Against the Final Diagnostic Outcome
Source: Front Allergy. 2021 Nov 23;2:765029. doi: 10.3389/falgy.2021.765029 (PMC8974758; doi:10.3389/falgy.2021.765029)
Supplement: Supplementary file 1 [file Data_Sheet_1.docx]

**Appendix**

**Allergy-Focussed Diet History – Adult Form**

Study No:

| **1** | **How old were you on your last birthday? _____________________**  **Gender:**  Male Female Prefer not to say  **Ethnicity:** White // Black // Mixed race// Asian // Chinese// Other // Prefer not to say  **Height** if known)______________**Weight** (if known) _____________ | | | |
| --- | --- | --- | --- | --- |
| **2** | **Your symptoms** (Tick box and circle relevant symptoms)   - **Skin**: flushing/redness, general itch, hives, swelling, eczema   **Pattern** - comes and goes (daily/weekly/monthly) OR continuous     - **Mouth/throat –** itching, swelling (lips, tongue, throat), hoarse voice, throat closure   **Pattern** - comes and goes (daily/weekly/monthly) OR continuous     - **Gastrointestinal**: Abdominal pain, nausea, vomiting, diarrhoea, blood in the stool, acid reflux or difficulty in swallowing   **Pattern** - comes and goes (daily/weekly/monthly) OR continuous     - **Eyes, nose and chest**: conjunctivitis, nasal itching, sneezing, cough, chest tightness, wheeze, shortness of breath   **Pattern** - comes and goes (daily/weekly/monthly) OR continuous     - **Heart**– dizziness, low blood pressure, rapid heartbeat, collapse   **Pattern** - comes and goes (daily/weekly/monthly) OR continuous     - **Anaphylaxis –** multi-system involvement e.g. skin symptoms plus chest or heart symptoms, or two or more symptoms from different symptom categories - **Other –** Looking pale, feeling tired, not eating, weight loss or other condition:   **Pattern** - comes and goes (daily/weekly/monthly) OR continuous | | | |
| **3** | How old were you when the symptoms first appeared? _______________________  In what circumstances did symptoms first appear? (During or following a meal, location etc.)  _____________________________________________________________ | | | |
| **4** | Where have your reactions taken place?  At home at work restaurant takeaway other location | | | |
| **5** | Please tick all of the remedies used in any of your allergic reactions and also state whether they were helpful   - Drank some water……………………Helpful? Yes No - Took an antihistamine ………………Helpful? Yes No - Took steroids………………………. Helpful? Yes No - Gave yourself adrenaline…………… Helpful? Yes No | | | |
| **6** | When you had the reaction, were any of the factors below also present? If so tick all that apply:  □Taking exercise □Drinking alcohol □Taking Aspirin or Ibuprofen  □Feeling tired or stressed □Start of menstruation □Unwell with an infection | | | |
| **7** | Do you have any of the following either now or when you were a child? Please tick all that apply   - ***Asthma***   How old were you when it started…………… Do you still have it now: Yes No  Medication …………………. Symptoms: mild/moderate/severe     - ***Hay fever***   How old were you when it started…………… Do you still have it now: Yes No  When do you get symptoms (circle all that apply)  Spring Summer Autumn Winter All Year Round  Medication……………………… Symptoms: mild/moderate/severe   - ***Food allergy in childhood***   How old were you when it started…………… Do you still have it now: Yes No   - ***Eczema***   How old were you when it started…………… Do you still have it now: Yes No  Medication………………………….. Symptoms: mild/moderate/severe   - ***High blood pressure***   Yes No If yes, which medication do you take for this…………………………   - ***Other ongoing medical condition***   Yes No If yes, please describe______________________________________ | | | |
| **8** | Do any of your immediate family (parents, brothers, sisters) have any of the following conditions:  Asthma hay fever food allergy eczema | | | |
| **9** | Do you have any allergies to rubber gloves, condoms or get wheezy blowing up balloons?  Yes No | | | |
| **10** | Have you had any allergy tests before? Yes No    If Yes please circle any foods or aeroallergens you had previous positive tests to:  Grass Trees Weeds Cat Dog House Dust Mite Moulds  Milk Egg Fish Shellfish Wheat Nuts Peanuts Sesame seeds | | | |
| **11** | Are you taking any regular medicines? Yes No  If Yes please list _________________________________________ | | | |
| **12** | | Are you taking any nutritional supplements? Yes No  If Yes please list _________________________________________ | | |
| **13** | | Please describe the meal/food involved in your most recent reaction: | | |
|  | | Please circle the symptoms you experienced  Mouth/throat: itching, swelling, difficulty in swallowing  Eyes/Face: swelling, flushing,  Skin: flushing, rash, hives (red itchy bumps on the skin)  Gut: nausea, vomiting, diarrhoea  Chest: short of breath, feeling wheezy, chest tightness, difficulty breathing,  Heart: feeling dizzy, feeling faint, | | |
|  | How soon did these symptoms appear after eating?  During the meal or After eating If after eating, how long to first symptoms:  5-10 mins 20-30 mins, 30-60 mins, 1-2 hours, 2-4 hours, more than 4 hours | | | |
| **If you have only had one reaction please got to Q14.**  **If you have had more than one reaction** - please give details of up to two other reactions, including the most severe) on the next page. | | | | |
|  | Foods Involved: | | | |
|  | | Please circle the symptoms you experienced  Mouth/throat: itching, swelling, difficulty in swallowing  Eyes/Face: swelling, flushing,  Skin: flushing, rash, hives (red itchy bumps on the skin)  Gut: nausea, vomiting, diarrhoea  Chest: short of breath, feeling wheezy, chest tightness, difficulty breathing,  Heart: feeling dizzy, feeling faint, | | |
|  | How soon did these symptoms appear after eating?  During the meal or After eating If after eating, how long to first symptoms:  5-10 mins 20-30 mins, 30-60 mins, 1-2 hours, 2-4 hours, more than 4 hours | | | |
|  | Foods Involved: | | | |
|  | | Please circle the symptoms you experienced  Mouth/throat: itching, swelling, difficulty in swallowing  Eyes/Face: swelling, flushing,  Skin: flushing, rash, hives (red itchy bumps on the skin)  Gut: nausea, vomiting, diarrhoea  Chest: short of breath, feeling wheezy, chest tightness, difficulty breathing,  Heart: feeling dizzy, feeling faint, | | |
|  | How soon did these symptoms appear after eating?  During the meal or After eating If after eating, how long to first symptoms:  5-10 mins 20-30 mins, 30-60 mins, 1-2 hours, 2-4 hours, more than 4 hours | | | |
| **14** | | Have you cut out any foods from your diet Yes No  If Yes - did cutting them out help? Yes No | | |
| **16** | | Please tick avoid column for any food group you are not eating and state why you are avoiding and exactly which foods in the group are being avoided | | |
|  | | | **Avoid?**  **Yes or No** | **Why** (allergy, intolerance, vegan, vegetarian, religious reason)**, Which** (all in group or just some**), What** (all forms including traces, raw only, cooked and raw, cooked only) |
| **Milk -**  milk, cheese, butter yoghurt | | |  |  |
| **Egg** | | |  |  |
| **Red meat, chicken** | | |  |  |
| **Fish** e.g. cod, salmon, trout | | |  |  |
| **Shellfish** – e.g. prawns, mussels, squid | | |  |  |
| **Tree nuts -** Hazelnut, almond, brazil nut, walnut, pecan, cashew, pistachio, macadamia | | |  |  |
| **Peanuts** | | |  |  |
| **Soy** **and other legumes**: chickpeas, peas, beans, lentils, lupin | | |  |  |
| **Seeds** - sesame, sunflower, pumpkin, poppy, mustard, pine nuts | | |  |  |
| **Fruit** e.g. apple, kiwifruit, peach, strawberry, banana, mango, avocado | | |  |  |
| **Fresh vegetables and vegetable juices**  e.g. tomato, carrot, celery | | |  |  |
| **Herbs and spices**  e.g. coriander, parsley, chilli, cumin, paprika, mustard | | |  |  |
| **Cereals -** Wheat, rice, barley, oats, corn, rye, spelt, quinoa | | |  |  |
| **Buckwheat** | | |  |  |
| **Drinks -** Cordial/squash, fizzy drinks, fruit or vegetable juices, smoothies, protein shakes | | |  |  |
| **Alcohol –** wine, beer, spirits | | |  |  |

**Patient 7-Day dietary assessment Date:**

**Age: Height: Weight:**

**Patient number:**

|  | Food / Type | Portion size / How often |
| --- | --- | --- |
| Food normally consumed at **breakfast**? |  |  |
| Food consumed at **midday**? |  |  |
| Food consumed at **dinner**? |  |  |
| Food **after dinner**? |  |  |
| Do you consume **fruits**?  **Vegetables?** |  |  |
| **Red meat?** (beef, lamb, veal)  **Organ meat?** (liver, kidney, heart)  **Pork**?  **Chicken**?  **Fish?** (fresh, canned in oil or brine)  **Eggs?** (cooking method) |  |  |
| Do you consume **pasta?**  Wheat, Wholegrain, white?  **Rice?** Brown, white?  **Oats?**  **Bread?** Wholegrain, brown, white?  **Potatoes?**  Other sources of CHO (corn) |  |  |
| **Lentils?**  **Pulses?** (chickpeas, dry beans)  **Nuts and seeds?**  **Beans?** |  |  |
| **Dairy products?**  **Milk?** (whole, semi-skimmed, skimmed)  **Cheese?** (Hard/soft)  **Butter?** |  |  |
| **Snacks throughout the day?**  **Chocolates? Cookies, cakes?**  **Sweets?**  **Sugar, honey?** |  |  |
| **What do you drink throughout the day?**  **Coffee?**  **Tea?**  **Fruit juice?**  **Soft drinks?** |  |  |
| **Alcohol?** |  |  |
| **Does this vary between week-days and weekends?** |  |  |
| **Spreads used?** (Butter, margarine, pro-active, cream cheese)  **Oil used for cooking** (OO, sunflower oil, coconut oil, rapeseed oil, butter) |  |  |
| **Do you purposefully avoid certain foods?** |  |  |
| **Do you eat out? / Order Takeaway?** |  |  |
| **Do you take any supplements?** |  |  |
